# Supplementary material for: Epigenetic age and lung cancer risk in the CLUE II prospective cohort study
Source: Aging (Albany NY). 2023 Feb 7;15(3):617–29. doi: 10.18632/aging.204501 (PMC9970317; doi:10.18632/aging.204501)
Supplement: Supplementary Tables [file aging-15-204501-s001.pdf]

SUPPLEMENTARY TABLES

Supplementary Table 1. Odds ratios and 95% confidence intervals for the association between epigenetic age acceleration (using 3 different measures) and the risk of lung cancer in the CLUE II study, stratified by age and smoking status.

	OR (95% CI)*	*p*-value	OR (95% CI)*	*p*-value
	Age < 65 years		Age ≥ 65 years	
AA_Hannum	0.63 (0.44, 0.90)	0.011	0.90 (0.53, 1.52)	0.69
AA_Horvath	0.62 (0.44, 0.88)	0.007	1.10 (0.69, 1.75)	0.70
AA_Pheno	0.76 (0.58, 1.00)	0.046	1.07 (0.75, 1.52)	0.72
IEAA_Hannum	0.69 (0.48, 1.00)	0.05	0.91 (0.52, 1.59)	0.74
IEAA_Horvath	0.73 (0.52, 1.04)	0.08	1.08 (0.67, 1.74)	0.74
IEAA_Pheno	0.74 (0.55, 0.98)	0.035	1.13 (0.77, 1.67)	0.52
	Current smoker**		Former smoker**	
AA_Hannum	0.76 (0.49, 1.20)	0.24	0.69 (0.44, 1.07)	0.09
AA_Horvath	0.68 (0.45, 1.02)	0.06	0.82 (0.54, 1.27)	0.38
AA_Pheno	0.93 (0.68, 1.28)	0.66	0.84 (0.61, 1.16)	0.30
IEAA_Hannum	0.93 (0.58, 1.48)	0.76	0.71 (0.45, 1.12)	0.14
IEAA_Horvath	0.81 (0.53, 1.22)	0.31	0.91 (0.58, 1.43)	0.69
IEAA_Pheno	1.02 (0.73, 1.44)	0.90	0.85 (0.60, 1.20)	0.36

Abbreviation: IEAA: Intrinsic epigenetic age acceleration. *Conditional logistic regression models, adjusting for batch effects, BMI, and smoking predicted packyears (using DNA methylation). **Never smokers were not included in this analysis are there were too few.

Supplementary Table 2. Correlations between epigenetic clocks and selected immune cell proportions (estimated from methylation data).

	AA Hannum	IEAA Hannum	AA Horvath	IEAA Horvath	AA PhenoAge	IEAA PhenoAge
**CD4 naïve**	−0.3677	−0.1935	−0.1884	−0.0011	−0.2249	−0.1104
***p* values**	<0.0001	0.0051	0.0066	0.9877	0.0011	0.1124
**CD8 naïve**	−0.4245	−0.4076	−0.2305	−0.1706	−0.32	−0.3196
***p* values**	<0.0001	<0.0001	0.0008	0.014	<0.0001	<0.0001
**CD8 memory**	0.3316	0.1264	0.3202	−0.0026	−0.0203	0.098
***p* values**	<0.0001	0.0689	<0.0001	0.9706	0.7713	0.1591
**B cell naïve**	−0.2507	−0.1485	−0.1786	−0.0609	−0.3272	−0.1856
***p* values**	0.0003	0.0323	0.01	0.3831	<0.0001	0.0073

Correlations for CD4 memory and B cell memory are not shown as the correlations were statistically significant. Abbreviation: IEAA: Intrinsic epigenetic age acceleration.
